# Antivaccinationist Candour

**Published:** 1808-05

**Authors:** Rees Price

**Affiliations:** Blackfriars Road


### To Dr. BRADLEY.

SIR,

IN a Number of your Journal, some time ago, I saw and approved a resolution, that you would no longer admit Communications respecting Vaccination, except under peculiar circumstances; I am not therefore, Sir, at present, about to offer any thing *for* or *against* the general principles of it, as I believe its merits are well established in the minds of all liberal and scientific men, whose experience has best entitled them to form an opinion on the subject; I am desirous only of expressing my marked reprobation of some of the vile means used by the Antivaccinists to affect its credit with the public, and also to state that they have had the temerity, by using my name, to make me appear as an abettor of their plans.

Some

Some time since, one William Howard, whose wife and some part of whose family I had attended in the small-pox, came to me, requesting my opinion of a letter which he intended to write to the Jennerian Society, the accuracy and tendency of which I saw much reason to disapprove: I consequently endeavoured to dissuade him from his purpose, and recommended that he should *state* the particulars; as it was my opinion, that those parts of his family, who had the small-pox when I attended them, had never been vaccinated, Mrs. Howard herself having declared to me, that her arm had never inflamed, neither were there any marks on any of their arms that I could discover; but on the arm of one boy, who escaped the small-pox, there was proof enough that Vaccination had taken effect. I gave him such advice, concluding that the Society would set him right in the affair, as he seemed a decent man, and had been a parish clerk. Instead of following my advice, however, I understood he did write to the Society, threatening, in case he did not hear from them soon, that it was his intention to publish the letter in the Newspapers. Now, in my mind, it requires no great stretch of imagination to conceive how he, William Howard, might have been prevented from putting this threat into execution! But judge, Sir, of my surprize and indignation, on finding my name in circulation in the Publican's Advertiser, attached to a vile unfeeling attack on Dr. Jenner, as the cause of the death of his wife by the small-pox, because an unsuccessful attempt had two years ago been made to protect her against this scourge of mankind by vaccination, and that too performed against his and her own inclination and conviction, by matter which had been previously immersed in hot water.

Permit me now to ask, why it never entered the head of this good man, to endeavour to protect his wife, who was by no means a young woman, from the hazard of the small-pox, before the period at which she was attempted to be vaccinated? Or, would *he*, provided that attempt had not been *forced* upon him contrary to his inclination, have had her at *that*, or any subsequent period, inoculated for the small-pox? When he has answered these questions, perhaps it may appear that Dr. Jenner had no more to do with the death of his wife, than yourself, Sir, who may now, for the first time, hear of it through the medium of this communication.

Farther, I as much believe that the man, William Howard, wrote the letter in the Publican's Paper as that I

wrote wrote it myself!' I have been assured, that between the death and burial of his wife, he, by representing the matter in his own way, collected a sum of fifteen pounds in the neighbourhood in which he then resided. Thus finding it a lucrative game, he thought it worth while to play for a larger stake, and publish the letter above alluded to, but not before (if report can be credited) he had had more than one interview with his friend Dr. Moseley.

Not one of the least circumstantial proofs in my possession, tbat William Howard did not write the letter in question, is, that he could entrust his wife, for a great length of time, to the care of a man who calls himself Dr. B*****, and has it on his door in conspicuous gilt letters, but of which, I hope, the Royal College of Physicians will take notice. By the following specimen of Dr. B's literature, we may judge first, how capable he (Dr. B.) must be to conduct disease; and, secondly, what discrimination William Howard must possess, to give such a man several fees for attempting to cure his wife of a decline, and then, on his failing to do so, that he should have no more wit than to wish for them back again. But Dr. B's letter to Wm. Howard will best explain the matter.

"Aprel 2nt 1807

"Mr Howard

"I undor Stand you wis for your Money Back My Attendants & Meadsons cums too Dubel the Som & If youor Inpottents Anny mor by Sand Lett of thatening I shell Dew My Salf the Plastre of Serving you with Anaction Booth for Sum and for Damegges Dount you think you got a Curtary Fool to Dial with Now I giv you Notic the Furst Scandil I heor From you I will Spond 20 Gunais butt Ill Make anuxampil of you

"Dr. B*****"

Now, Sir, I am only surprised, from the long declining state of Mrs. Howard's health, and from the long attendance of Dr. B. that she, poor woman, should have lived so long as at last to die of the small-pox on the 12th of February, 1808.

I own, I have little time, inclination, or talents for investigations of this nature; but now that I am on the subject, I will endeavour to collect a few farther notions, which have long been floating in my mind; nor do I think I can better describe them, than by imagining the reception I should in all probability have met with from Doctor Jenner, had I gone and stated to him, what I now take take the liberty of writing to you. He, perhaps, with all that urbanity so peculiar to himself, would have said,—"My dear friend, I feel much obliged for the kind trouble you have taken on this occasion; but I feel satisfied myself with having stated and explained to the public one of the greatest blessings it has pleased Providence to bestow on mankind; nor do I think the public ought to expect from me, to answer every base attack on Vaccination, in Newspapers or in Debating Societies. Depend on this, Sir, it is not from its enemies that Vaccination has any thing to apprehend; if you could prevail on some of its friends to use a little less zeal in its support, and particularly, if you could prevent one gentleman from exposing himself and the cause, by defending it, in his way, in a Debating Society, where William Howard and other objects, with the sole view of extorting Charity, never fail to attend, you would do a real service. If, Sir, I say, you could but defend me from the intemperate zeal of some of the Friends of Vaccination, I am sure I am more than a match myself for all its Enemies."

> "An open Foe may prove a Curse,
> But an ILL-JUDGING Friend is worse."

Thus, Sir, I have thought it right, by these means, to show the Public that I am better satisfied with Vaccination, than with the vile attempt which has been made to make it appear otherwise.

I am, Sir, &c.

REES PRICE.

*Blackfriars Road, April* 19, 1808.

